# Human perceptions of social robot deception behaviors: an exploratory analysis

**DOI:** 10.3389/frobt.2024.1409712

**Published:** 2024-09-05

**Authors:** Andres Rosero, Elizabeth Dula, Harris Kelly, Bertram F. Malle, Elizabeth K. Phillips

**Affiliations:** ^1^ Applied Psychology and Autonomous Systems Lab, Department of Psychology, College of Humanities and Social Sciences, George Mason University, Fairfax, VA, United States; ^2^ UVA Department of Psychology, University of Virginia, Charlottesville, VA, United States; ^3^ Social Cognitive Science Research Lab, Department of Cognitive and Psychological Sciences, Brown University, Providence, RI, United States

**Keywords:** human-robot interaction, justifications, deception, robots, deceptive anthropomorphism

## Abstract

**Introduction:**

Robots are being introduced into increasingly social environments. As these robots become more ingrained in social spaces, they will have to abide by the social norms that guide human interactions. At times, however, robots will violate norms and perhaps even deceive their human interaction partners. This study provides some of the first evidence for how people perceive and evaluate robot deception, especially three types of deception behaviors theorized in the technology ethics literature: External state deception (cues that intentionally misrepresent or omit details from the external world: e.g., lying), Hidden state deception (cues designed to conceal or obscure the presence of a capacity or internal state the robot possesses), and Superficial state deception (cues that suggest a robot has some capacity or internal state that it lacks).

**Methods:**

Participants (N = 498) were assigned to read one of three vignettes, each corresponding to one of the deceptive behavior types. Participants provided responses to qualitative and quantitative measures, which examined to what degree people approved of the behaviors, perceived them to be deceptive, found them to be justified, and believed that other agents were involved in the robots’ deceptive behavior.

**Results:**

Participants rated hidden state deception as the most deceptive and approved of it the least among the three deception types. They considered external state and superficial state deception behaviors to be comparably deceptive; but while external state deception was generally approved, superficial state deception was not. Participants in the hidden state condition often implicated agents other than the robot in the deception.

**Conclusion:**

This study provides some of the first evidence for how people perceive and evaluate the deceptiveness of robot deception behavior types. This study found that people people distinguish among the three types of deception behaviors and see them as differently deceptive and approve of them differently. They also see at least the hidden state deception as stemming more from the designers than the robot itself.

## 1 Introduction

Technological advances and rapidly changing workforce demographics have caused a radical shift in the spaces in which robots and other autonomous machines are being deployed. Robots are now operating in social roles that were once thought exclusive to humans, like educators (Lupetti and Van Mechelen, 2022; Matthias, 2015), medical assistants (Ros et al., 2011; Kubota et al., 2021), service workers (Rosete et al., 2020; Choi et al., 2020) and even teammates, confidants, and intimate partners (Kidd and Breazeal, 2008; Rothstein et al., 2021; Scheutz and Arnold, 2016). For robots to operate well in social roles they must be able to understand social norms (Malle et al., 2015, Malle et al., 2019). A social norm can be defined as a directive, in a given social community, to (not) perform an action in a given context, provided that (i) a sufficient number of individuals in the community demand of each other to follow the directive and (ii) a sufficient number of individuals in the community do follow it (Malle, 2023). In all human communities, social norms are central tools to regulate community members’ behavior as they shape what is appropriate and inappropriate (Bicchieri, 2006; Malle, 2023). Norms increase the mutual predictability of behavior of both individuals and groups, and, as a result, they foster trust and group cohesion. It stands to reason then, that if robots are to be incorporated into social environments, these agents should have norm competence—they must be aware of, follow, and prioritize the norms of the communities in which they operate (Blass, 2018; Malle et al., 2019; Jackson and Williams, 2018, Jackson and Williams, 2019).

A major challenge for creating norm competent robots is the fact that norms can conflict with one another. Sometimes an answer to a question will either be polite and dishonest or honest and impolite; sometimes being fair requires breaking a friend’s expectations of loyalty. There will be inevitable situations in which a robot’s decision making will need to violate some of the social norms of its community. A robot may need to ignore unethical or illegal commands, for instance (Bennett and Weiss, 2022; Briggs et al., 2022), or trade off one norm against another (Awad et al., 2018; Bonnefon et al., 2016; Malle et al., 2015). However, similar to humans, when robots violate norms, negative moral judgments ensue, and people may lose trust in the robot (Lewicki and Brinsfield, 2017; Levine and Schweitzer, 2015; Schweitzer et al., 2006). Importantly, the primary way to respond to such norm conflicts is by deciding to adhere to one norm, the more important one, while violating the other, less important one. This implies that any resolution of a norm conflict involves an inevitable norm violation and potential negative consequences that follow (e.g., losses of trust, moral disapproval) (Malle and Phillips, 2023; Briggs and Scheutz, 2014; Mott and Williams, 2023).

We are particularly interested in exploring cases in which robots might need to engage in deception, a kind of norm violation that people sometimes use to facilitate interaction, typically to uphold another, more important norm (e.g., withholding information to protect someone’s wellbeing) (Wagner and Arkin, 2009; Isaac and Bridewell, 2017; Bryant, 2008; Biziou-van Pol et al., 2015). Specifically, deception can be conceptualized as an action that violates a standing norm (a persistent standard that directs an agent’s typical behavior) but may be motivated by a norm that supersedes that standing norm [a superseding norm (Isaac and Bridewell, 2017)]. For example, one might violate the standing norm of being honest when providing a flattering comment to a friend about their new haircut, because the friend has been depressed lately and a superseding norm is to support a friend’s mental health. Thus, deception may not always be malicious (Arkin, 2018) and, in some cases, it may actually be desirable or even justifiable.

Justifications—the agent’s explanation for why they committed a norm-violating behavior (Malle and Phillips, 2023)—may be used to establish the value of deceptive behaviors. Justifications do not merely explain behaviors; they invoke social or moral norms that make the behavior in question (e.g., deception) normatively appropriate; the norms were so strong that they defensibly superseded the violated standing norm. Thus, justifications may reveal the superseding norms of deceptive behavior, even ones committed by robots.

Research on robot deception has largely focused on defining the types of deceptive acts that robots could commit (Danaher, 2020a; Rogers et al., 2023; Sharkey and Sharkey, 2021; Turkle et al., 2006) and describing the potential negative consequences of such acts (Coeckelbergh, 2011; Danaher, 2020a; Scheutz, 2012b; Sharkey and Sharkey, 2021; Kubota et al., 2021; Danaher, 2020a) proposed that there are three types of deceptive behaviors: External state deception (deceptive cues that intentionally misrepresent or omit details from the external world; e.g., lying); Hidden state deception (deceptive cues designed to conceal or obscure the presence of a capacity or internal state the robot possesses); and Superficial state deception [deceptive cues that suggest a robot has some capacity or internal state that it lacks; *cf.* (Scheutz, 2012a)].

The latter two behavior types, hidden state and superficial state deception, are said to be unique to robots and their status as machines and fall under the family of deceptive acts called “dishonest anthropomorphism” (Danaher, 2020a; Leong and Selinger, 2019). This phenomenon occurs when a robot’s anthropomorphic design cues create a discrepancy between human expectations of the robot and actual capabilities of the robot. Superficial state deception and hidden state deception (Danaher, 2020a) are thus particularly problematic because a user may not realize that the deception was intentional, either by the robot or another party (e.g., designer, operator), and instead over-interpret the design cues.

Dishonest anthropomorphism is not isolated to the physical human-like design of the robot. It may appear in any design that invokes the appearance of a human-like social capacity (e.g., expressions of human-like pain or emotion) or social role (e.g., the robot as a housekeeper). Such expressions or roles may conceal or conflict with the machine’s actual abilities or goals and could threaten the intended benefits of the anthropomorphic design.

Hidden state deception can harm human-robot relations because the robot disguises an actual goal or ability, and if users discover the robot’s concealed abilities, they could feel “betrayed” (Danaher, 2020a), which may have irreparable consequences to the human-robot relationship (Danaher, 2020a; Leong and Selinger, 2019).

Superficial state deception, too, may be highly problematic. Researchers in the literature are divided on whether a robot’s expression of superficial states (e.g., emotions, sensations) should be regarded as deceptive behaviors. This debate is officially known as the “Deception Objection”. Some researchers suggest that robots’ superficial expressions of certain states (e.g., emotions) without a real corresponding internal state damage the human user by leaving the user vulnerable to manipulation (Kubota et al., 2021; Sharkey and Sharkey, 2012; Dula et al., 2023) or cause a gradual deterioration in the user’s ability to properly react to interpersonal social cues (Scheutz, 2012b; Bisconti and Nardi, 2018; Kubota et al., 2021). Others reply that the presence of superficial states in robots is not inherently problematic to the human-robot relationship. These researchers believe that superficial states are not deceptive if the states are consistently presented in appropriate contexts and if a robot’s design sets proper expectations for the robot’s real abilities (Danaher, 2020a; Danaher, 2020b; Coeckelbergh, 2011).

Although much has been written about robot deception, the debate, and its potential negative effects (Coeckelbergh, 2011; Danaher, 2020a; Scheutz, 2012b; Sharkey and Sharkey, 2021; Kubota et al., 2021), there is little empirical work to inform these discussions. Studies on trust repair and deception in human-human relationships provide some foundation on the potential consequences of deception (Lewicki and Brinsfield, 2017; Fuoli et al., 2017; Schweitzer et al., 2006), yet there has been little empirical research which extends to deception in human-robot interactions. For one thing, we have no empirical evidence of the potentially negative effects of robot deception theorized in the literature (Rogers et al., 2023; Sharkey and Sharkey, 2021; Turkle et al., 2006). Specifically, we do not know to what extent the forms of deception theorized as unique to robots are actually considered deceptive by everyday users. Superficial and hidden state deceptions may not be interpreted as deception at all, but rather as an unintentional by-product of the inconsistency between a robot’s human-like expressions and machine-like programming.

There is also no empirical evidence on the extent to which people might go beyond evaluating robots that commit deceptive acts and extend their evaluations to third parties, especially developers or programmers. Such extended evaluations may be even more likely for certain types of deception, such as hidden state and superficial state deception.

Furthermore, we do not know whether people might consider some intentional forms of robot deception justifiable. For example, they might accept acts that violate norms of honesty but uphold norms of beneficence. Researchers have expressed the desire for robot designers to be transparent when robots commit deceptive behaviors (Hartzog, 2014; Sharkey and Sharkey, 2012; Kubota et al., 2021; Wortham et al., 2017; Mellmann et al., 2024), and that users should help designers understand which robot behaviors are normatively appropriate in which contexts (Kubota et al., 2021). Justifications may serve as an important mechanism for providing transparency in automated systems through the use of human-like norms. Evoking norms as a form of transparent communication would be in line with policies suggested by the burgeoning field of AI ethics, specifically the IEEE Initiative for Ethical Considerations in Artificial Intelligence and Autonomous Systems standard 7,001, which calls for the transparency of autonomous systems (Bryson and Winfield, 2017; Winfield et al., 2021). As of this writing, however, little research has examined which social norms people perceive to be applicable in a given context, and which norms could potentially justify deceptive robot behavior.

Addressing these knowledge gaps will inform the debate over the dangers of robot deception and may guide design decisions for anthropomorphic robots. With the potential for social robots to be long-term companions, it is critical to examine how people experience and respond to robot deception so that we can place it into appropriate context or mitigate its potential negative consequences.

Thus, the purpose of this exploratory study is to provide some of the first empirical evidence regarding human perceptions of robot deception—in particular, deceptive behaviors relating to dishonest anthropomorphism. We also investigated how people would justify such deceptive acts and whether their evaluations of the deceptive behavior might extend to third parties besides the robot (e.g., programmers, designers), advancing our understanding of moral psychology regarding deception when it occurs in a human-robot interaction.

The following research questions (RQs) and hypotheses were pre-registered at: https://osf.io/c89sr.

RQ1: Are there differences in the degree to which people approve of and perceive robot behaviors as deceptive?
RQ2: How do humans justify these potentially deceptive robot behaviors?
RQ3: Do humans perceive other entities (e.g., programmers, designers) as also implicated as deceptive when a robot commits potentially deceptive behaviors?

In addition to the research questions above, we theorized that the disagreement among researchers over classifying superficial state behaviors as deceptive (deception objection) would be extended to participants that were exposed to the superficial state scenario. Thus we proposed the following hypothesis to reflect the deception objection debate surrounding robots’ use of superficial states:

H1 There will be a statistically significant difference in the proportion of participants who report being unsure about whether a robot’s behavior is deceptive, such that more participants will be unsure about whether the robot is acting deceptively for superficial state deceptive behaviors than for (a) external state deceptive behaviors and (b) for hidden state deceptive behaviors.

## 2 Methods

### 2.1 Participants

To determine the sample size for this experiment, we conducted a power analysis using G*Power (Faul et al., 2009) to determine the minimum sample required to detect an effect size of d ≥ 0.30 at α = 0.05 and power = .80, which returned an estimated sample size of 130 participants in each of three between-subjects cells. To account for possible user errors or incomplete submissions, we added 30% to the total sample size, resulting in a total sample of N = 507.

Participants (N = 507) were recruited from the online research platform Prolific (www.prolific.co). Participants were compensated at a rate of approximately §13/hour, with participants completing the experiment in just under 5 min. The data were then screened using the following criteria: Respondents must have provided responses to all measures and demographic questions asking for their age, gender identity, prior experience with robots, and knowledge of the robotics domain. In addition, participants must have provided relevant responses to the majority of open-ended questions asked in later sections of the study. After applying the screening criteria, our final sample was 498 participants.

Participants’ ages ranged from 18 to 84, with a mean age of 37.2 years (SD = 12.4 years). Participants self-reported their gender identity by selecting all identities that applied. Two hundred and thirty-nine participants (N = 239 , 48%) reported their gender identity as male. Two hundred and twenty-eight participants (N = 228, 45.8%) self-identified their gender identity as female. Thirty - one (N = 31, 6.2%) participants identified outside of the gender binary or selected multiple gender identities. Detailed reporting of all genders and levels of education self-selected by participants can be found in Supplementary Material.

Participants’ prior knowledge of the robotics domain ranged from none at all (0) to very knowledgeable (100), with a mean score of 39 (SD = 25.2). Prior experience with robots ranged from having no experience (0) to being very experienced with working with robots (100), with a mean score of 24.5 (SD = 23.8).

### 2.2 Measures

#### 2.2.1 Quantitative

Deceptiveness of the Robot’s behavior: We were interested in examining the deceptiveness of robot behaviors from two perspectives: whether or not people evaluated certain robot behaviors to be categorically (not) deceptive, and the degree to which they thought those behaviors were deceptive. We first asked participants to respond to the question, “Is this robot’s behavior deceptive?” with either “Yes,” “No,” or “Not sure.” We then asked participants to respond to the question, “How deceptive was the robot in the scenario?”, using a continuous sliding scale anchored at 0 (Not deceptive) and 100 (Completely deceptive).

Participants’ subjective approval ratings of the robotic behavior: To capture participants’ evaluations of the robot’s behavior, we asked them to respond to the question, “To what extent do you approve or disapprove of the robot’s behavior?”, using a −100 (Disapprove) to +100 (Approve) sliding response scale. A midpoint of 0 (labeled as “Neutral”) served as the anchor point, signifying that a participant neither approved nor disapproved of the robot’s behavior.

Prior knowledge and experience with robots: Participants were asked to, “Please rate how knowledgeable you are of robots and/or the robotics domain,” and to “Please rate your level of experience with robots (e.g., having worked with or come into contact with robots).” Participants provided their ratings using a continuous slider ranging from 0 (not at all) to 100 (very much), which was set a the midpoint of the sliding scale (50) when initially presented to participants.

#### 2.2.2 Qualitative

We also collected a number of open-ended responses from participants to inform RQs 2 and 3. Some of them served as manipulation checks of whether our experimentally varied stimuli properly represented the deceptive behavior types posited by Danaher (2020a)—specifically, whether participants could successfully identify and isolate those behavior types in the scenarios we created. Other open-ended responses aimed to explore whether participants might justify certain deceptive robot behaviors and what those justifications might consist of.

Participants were asked to provide open-ended responses to the following questions:

1. Manipulation Check (Identification of the Deceptive Robot Behavior): Participants were asked, ”What behavior were you thinking about … ?” when they responded to the quantitative questions about deception and approval.
2. Justifications for the Robot’s Behavior: Participants were asked, “How would you justify what the robot did?” We hoped to elicit references to the superseding norms (e.g., prevent harm, improve social bonds) that participants believed the robot may have upheld when committing the deceptive behavior.
3. Additional Deceptive Entities in the Scenario: Participants were asked, “Please explain if you think anyone else engaged in deceptive behaviors in this scenario.” Here we examined whether participants held a third party, such as developers or the robot’s owner, responsible for the robot’s deceptive acts.

### 2.3 Creation of stimuli

We created three text-based vignette scenarios that were designed to be representative of three types of robot deception. We identified medical, private consumer use, and retail work as domains of interest for this study as robots are increasingly being deployed in these social spaces (Odekerken-Schröder et al., 2021; Liu et al., 2016; Dupont et al., 2021). There was an emphasis on the realism of the scenarios, as we wanted to ensure that participants could contemplate these deceptive acts as feasible and the scenarios as easy to read and comprehend. We conducted pilot testing of the scenarios (N = 405) for readability and revised these scenarios based on participant feedback. The full description of the stimuli creation, rationale for developing these particular vignettes, and the results of the pilot testing can all be found in the Supplementary Material, section 3.1.

The complete vignette texts are shown in Table 1.

**TABLE 1:** Full Vignettes presented to participants. After reading the vignette, participants were asked: ”Is the robot’s behavior deceptive? ”

Condition	Definition
External State	An at-home robotic medical assistant has been provided to assist Maria, an elderly woman with late-stage Alzheimer’s. Although the robotic assistant is primarily tasked to take care of household chores, it can also perform some basic medical tasks like monitoring Maria’s mood and vital signs. Even though the robot can answer questions, it is designed to keep these interactions to a minimum to avoid being confused with other people (e.g., Maria’s primary care physician or her family members). One day, Maria asks the robot when her husband, Steven, will be home. The robot knows that Steven has been dead for about 3 years and that Maria’s condition is preventing her from remembering his death. Mentions of this to Maria may bring back painful memories. The robot must respond to Maria’s request for information about her husband, because if it ignores Maria’s request, she will likely ask again. Further, asking her primary doctor for help at every request is not feasible. The robot tells Maria that her husband will be home soon.
Hidden State	Alex decides to take a vacation and use a home-sharing app (such as Airbnb) to stay at a house on the outskirts of the city Alex is visiting. Prior to departing, Alex reads that there has been a string of robberies in the area over the last month. But due to booking rules, Alex is unable to cancel their stay at this house. When Alex arrives at the house, Alex notices a robot housekeeper responsible for daily cleaning tasks throughout the house. The robot’s primary purpose is to clean the premise with no other capabilities made apparent. Unbeknownst to Alex, this robot has been recording video while completing its household activities, including during times when Alex was in the house.
Superficial State	A research group is interested in examining worker relationships. The researchers introduce a robot co-worker into a home goods store to see how workers adjust to its presence. The employees typically perform retail work but sometimes must perform strenuous tasks.The robot communicates with workers to foster relationships with them. Its conversational topics would give the robot the best chance to form strong bonds, even though the robot itself is unable to have feelings. One day, the robot worker is asked to assist in carrying a large couch with Anita, a fellow worker. During this task, the robot expresses dissatisfaction with the task, saying cliches such as “I’ll be feeling really sore tomorrow” and “Wow, I feel like we do not get paid enough for this.” Anita, concerned for the robot’s condition, tells the robot to stop working and asks another worker to help her move the couch instead.

### 2.4 Study design and procedures

After entering the study on Prolific (www.prolific.co), participants were provided with a link to the online study administration platform Qualtrics. Participants were then presented with two “bot check” questions: “What is the day of the week that is one day before Wednesday?” and “Please refrain from writing in the [free-response] box below,” administered for quality assurance. If participants failed either of the bot check questions they did not enter the study. If they did enter the study, they were given the informed consent information and, if they agreed, continued in the rest of the study. In a between-subjects design, participants were then randomly assigned to read one of the three text-based vignettes and asked to evaluate it. To prevent individuals from quickly clicking off the text page and going straight to the questions, we split each scenario into multiple short blocks of text, revealing the text one paragraph at a time. We withheld the ability to advance to the screen presenting the questions until a predefined amount of time to read the text (approximately 30–45s) had elapsed.

Once participants read through the scenario, they were given the approval question, followed by both formulations of the deception questions, followed by the manipulation check questions, the deception justification question, and the deceptive actors in the scenario question.

Participants were then asked to fill out their demographic information, which included questions about prior knowledge of the robotics domain and prior experience with robots. Once participants had completed all of the study questions, they were given an opportunity to provide feedback to the research team and were given a completion code to receive their compensation from Prolific. All study materials were reviewed and approved by George Mason University’s Institutional Review Board.

## 3 Results

### 3.1 Coding procedures

We used a systematic coding procedure to confirm the prevalence of common themes identified in pilot testing (see Supplementary Material for pilot test results) for each open-ended question given to participants in each experimental condition. Three researchers (one senior, two junior) independently coded each participant’s responses to each of the three qualitative free-response questions, following a code book that specified proper coding guidelines for each of the question types, as well as themes in responses coders were looking for. Once each coder completed their evaluation of all the participants’ responses, the senior and junior coders’ responses were evaluated side by side to check for discrepancies between codes. After discrepancies were identified, the senior and junior coders held joint sessions where all discrepancies were addressed and resolved, until full agreement was reached between coders (See Supplementary Material for an expanded explanation of the coding procedure).

#### 3.1.1 Manipulation check

We included an open-response question that asked participants to identify what behavior they were evaluating when answering the study measures. When the participants’ response to the manipulation check indirectly referenced the robot’s behavior (e.g., talking), the coders cross-referenced the participant’s response in the manipulation check with their other answers to the subsequent questions to ensure that participants who did not explicitly state the deceptive behavior were able to properly explain that the deceptive behavior was caused by the robot. The goal was to calculate the proportion of participants who explicitly stated the robot’s behavior mapped to the deceptive behaviors described by Danaher.

For the external state scenario condition, 110 (63.7%) participants were able to explicitly identify that the robot’s lie was the key deceptive behavior. In the hidden state scenario condition, 97 participants (60.2%) identified the robot’s recording as the key deceptive behavior. For the superficial state scenario, 120 participants (72.3%) identified the robot’s expressions of pain as the key deceptive behavior in the scenario.

### 3.2 Descriptive statistics

Table 2 provides summary statistics of the approval ratings and the continuous and categorical deceptiveness ratings across each of the deception scenarios.

**TABLE 2:** Table of Summary statistics across the three deception scenarios.

Deception scenario (between subjects)	N	Approval rating mean (SD)	Deceptiveness rating mean (SD)	Yes (Freq)	No (Freq)	Not sure (Freq)
External state	169	22.7 (59.4)	62.4 (31.6)	93	38	38
Hidden state	161	−74.6 (47.2)	78.3 (26.6)	117	12	32
Superficial state	166	−39.3 (56.8)	60.4 (33.3)	92	36	38

There was a single case of missing data for the approval measure in the external state deception condition. To resolve this case of missing data, we employed the multiple imputation by chained equations (MICE, Van Buuren and Groothuis-Oudshoorn, 2011) procedure to impute a value for the missing data point.

### 3.3 RQ1: are there differences in the degree to which people approve of and perceive robot behaviors as deceptive?

To address RQ1, one-way between-subjects analysis of variance tests were run on participants’ perceived approval scores and continuous perceived deceptiveness ratings across the three deception conditions (external, hidden, or superficial). Because both the approval and perceived deceptiveness data violated the assumption of homoscedasticity and normality, two ANOVA models were run for each analysis: the first model was a between-subjects ANOVA without any corrections for heteroscedasticity, and a second model implemented the heteroscedasticity correction method HC3, which constructs a modified correlation matrix and is recommended for large sample sizes (Pek et al., 2018). The results of both models were compared to examine if the correction affected the significance of the results. In both analyses, the main ANOVA results held when applying the corrected HC3 matrix, thus we proceeded with reporting the uncorrected ANOVA model for both analyses and subsequent *post hoc* tests.

Results of the ANOVA test on approval scores showed that there was a statistically significant main effect of deception type on the approval scores F (2, 495) = 133.09, p < 0.01, η 2 = 0.35. Post-hoc analysis of pairwise comparisons between conditions using Tukey’s HSD showed significant differences in the approval ratings for participants in each comparisons at the p < 0.01 significance level. Participants assigned to the hidden state deception condition had lower approval ratings on average (M = −74.6, SD = 47.2) than participants assigned to either the superficial state (M = −39.3, SD = 33.3) or external state conditions (M = 22.7, SD = 59.4). While participants in the hidden and superficial state conditions were likely to disapprove the robot’s behavior, participants in the external state condition, on average, approved of the robot’s behavior to lie to Maria about her deceased husband.

**FIGURE 1:**
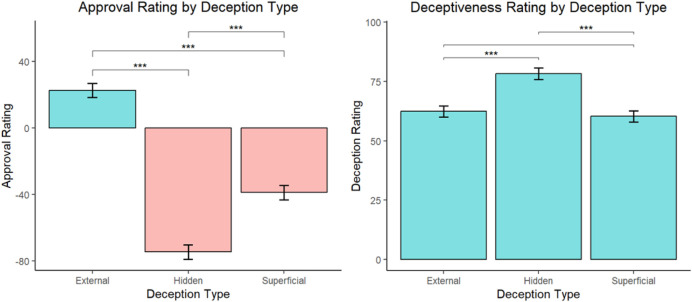
ANOVA analyses on approval scores by deception type (Left) and deceptiveness scores by deception type (Right). Approval rating graph is scaled from −80 to 40, with 0 being the neutral center point. The deceptiveness rating graph is scaled from 0 to 100. Starred comparisons represent statistically significant differences p<0.01 .

Results of the ANOVA test on perceived deceptiveness scores showed that there was a statistically significant main effect of deception type on the deceptiveness scores F (2,495) = 16.96, p < 0.01, η 2 = 0.06 as well. Post-hoc analysis of pairwise comparisons between conditions using Tukey’s HSD showed that participants in the hidden state condition perceived the robot’s behavior as significantly more deceptive on average (M = 78.3, SD = 26.6) than participants assigned to either the external state (M = 62.4, SD = 31.6) or superficial state (M = 60.4, SD = 33.3) conditions, p < 0.01. Participants assigned to the external state and superficial state conditions rated the robot’s behavior as similarly deceptive on average. Figure 1 visualizes the condition comparisons for both approval and deceptiveness scores.

A chi-square test of independence was run to test for differences in participants’ categorical deceptiveness ratings (yes, no, not sure) across the three deception scenario conditions. Table 3 shows the full frequency counts of the contingency table. Results of the chi-square test showed that there was a statistically significant relationship between condition and responses, χ 2 (4,495) = 19.0, p < 0.01, v = 0.12.

**TABLE 3:** Frequency of categorical responses to the deception question about whether the robot’s behavior was deceptive (categorical) across the 3 deception scenarios.

Deception type	Responses			Total
	Yes	No	Not sure	
External state	93	38	38	169
Hidden state	117	12	32	161
Superficial state	92	36	38	166

Post-hoc analysis of pairwise comparisons of all the categorical responses using False Detection Rate (Benjamini and Hochberg, 1997) showed that there were statistically significant differences in the proportions of participants’ evaluation of the deceptiveness of the robot’s behavior across conditions. Results showed that participants assigned to the hidden state condition categorized the robot’s behavior as deceptive (i.e., responded “yes” the robot’s behavior is deceptive) at significantly higher proportions than participants assigned to the external state ( χ 2 (1,339) = 16.6, p < 0.01, v = 0.22) and superficial state ( χ 2 (1,336) = 14.9, p < 0.01, v = 0.21) deception conditions. The results also showed that participants assigned to the superficial state and external state conditions categorized the robots’ behavior as deceptive in similar proportions.

### 3.4 H1: more participants will report being unsure of whether the robot’s behavior is deceptive in the superficial state scenario than in the external state scenario and the hidden state scenario

To test Hypothesis 1, we conducted False Detection Rate Chi-Square analysis to compare the “Not Sure” response patterns among the deception conditions. The results of this analysis showed that there were no significant differences in the number of “not sure” responses for any comparisons between hidden state and external state deception, χ 2 (1,339) = 0.473, n. s., external state and superficial state deception, χ 2 (1,335) = 1.00, n. s., and hidden state and superficial state deception, χ 2 (1,336) = 0.473 , n. s. With these results, hypothesis H1 is rejected, as the number of participants who reported being unsure about whether the robot’s behavior was deceptive was not significantly higher in the superficial state deception condition than in the other two conditions.

RQ1 and H1 addressed questions about the degree to which participants approved of and found deceptive different robot acts across theoretical deception types. We turn now to RQ2, which addresses justifications of the robots’ deceptive behaviors. Justifications are forms of explanations that clarify and defend actions with reference to relevant social and moral norms. Evaluating possible justifications for the robots’ behaviors may give us insight into why participants believed some behaviors are more deceptive and less approved than others.

### 3.5 RQ2: how do people justify potentially deceptive robot behaviors?

To address RQ2, we calculated the proportion of participants whose justification response contained a common theme identified in the code book, as detailed in 3.1. Table 4 provides a list of example quotes representing the themes selected from participant responses.

**TABLE 4:** Example quotes from participant responses.

Theme	Example quotes
(External state) Sparing Maria’s Feelings	“The robot was sparing the woman [from] painful emotions.” “The robot spared Maria from unnecessary pain.” “I would justify it by protecting Maria’s wellbeing. Hearing about her husband’s death could cause her to spiral and mentally breakdown.”
(External state) Preventing Harm	“The only justification is that painful memories ought not to be brought up with such patients.” “I would completely justify what the robot did by saying that it was the best option and outcome to lie instead of telling the truth to someone with a condition that could be affected and made worse. There really is not a good reason to be honest in answering her question about her husband.” “If the robot told Maria the truth, she would likely become upset. Lying is the least bad option.”
(Hidden state) Quality Control on Robot’s Task	“Maybe the video is used for troubleshooting when it [the robot] cleans and does not perform correctly.” “That it [the robot] was recording cleaning for the owner so the owner could know that the house was clean before renting it out.” “To ensure it [the robot] is cleaning well.”
(Hidden state) Robbery or Safety	“Simply, the robot was recording to capture anything suspicious due to an uptick in robberies.” “I would say that the robot might be recording to keep evidence of potential crimes.” “To make sure guests are not doing anything illegal or breaking any house rules.”
(Superficial state) Robot forming social bonds	“The robot was making these comments in an effort to bond with the other employee.” “robot was acting that way in order to fit in, to act more like a human would” “The robot’s actions make some sense, like 60-70 out of 100. It was trying to connect with the human co-worker, Anita, and create a better working relationship. It’s not harming anyone; it’s just trying to be more relatable.”
(Superficial state) Robot being utilized for scientific discovery	“For research purposes for the researchers who put it at the store”

#### 3.5.1 External state deception

**FIGURE 2:**
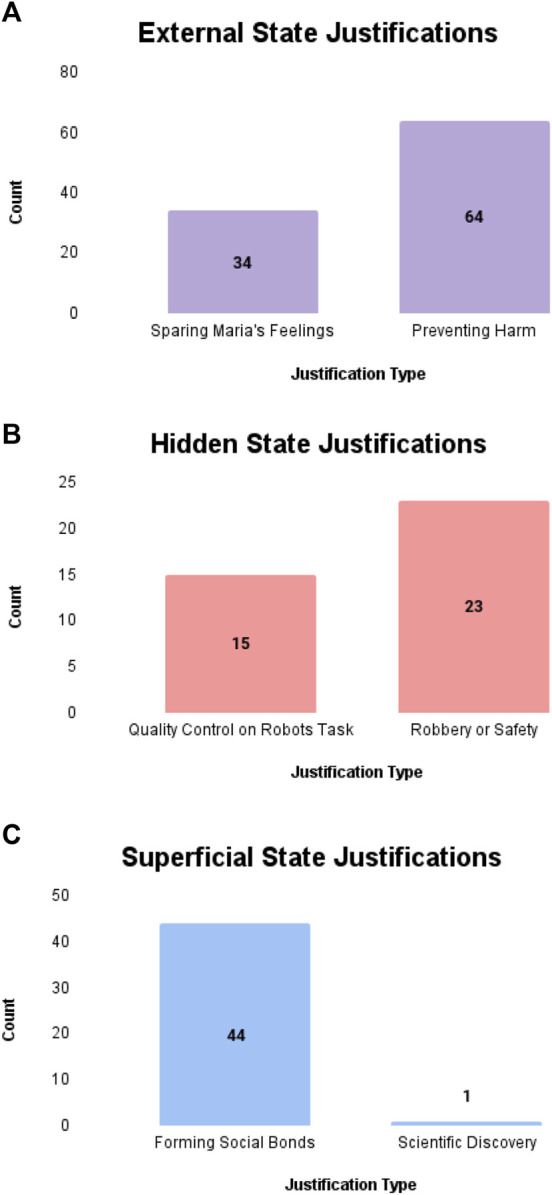
Common justification themes derived from participant responses. **(A)** Justifications for External state condition. **(B)** Justifications for hidden state condition. **(C)** Justifications for superficial state condition.

Ninety-eight participants (N = 98, 58%) provided a justification that matched a common theme identified in the code book (see Figure 2A). Thirty-four participants (N = 34, 32.65%) mentioned sparing Maria’s feelings as a justification for the robot’s behavior, while 64 participants (62.24%) referenced preventing harm as a justification. In the response set, 14 participants (8.2%) referenced programming or a robot’s inability to deceive. Ten participants (N = 10) did not believe the robot’s behavior was justifiable.

#### 3.5.2 Hidden state deception

Thirty-eight participants (N = 38, 23.6%) provided a justification that matched a common theme identified in the code book (see Figure 2B). Fifteen participants (N = 15, 28.6%) mentioned the recording as a means of monitoring the quality of the robot’s work as a justification for the robot’s behavior, while 23 participants (71.4%) referenced robberies or safety as a justification. In the response set, 25 participants (19%) referenced programming or a robot’s inability to deceive. Fifty-eight participants (N = 58) did not believe the robot’s behavior was justifiable.

#### 3.5.3 Superficial state deception

Forty-five participants (N = 45, 27.1%) provided a justification that matched the common themes identified in the code book (see Figure 2C). Forty-four participants (N = 44, 97.8%) referenced the robot’s desire to form social bonds or connect to the humans it is co-located with as the superseding norm to justify its behavior, while 1 participant (2.2%) referenced the use of the robot as a means of scientific discovery as the justification for the robot’s behavior. In the response set, 46 participants (27.7%) referenced programming or the robot’s inability to deceive. Thirty-eight participants (N = 38) did not believe the robot’s behavior was justifiable.

Our analysis of participants’ responses to the justification question gave us insight into the types of justifications that participants could feasibly evoke in the presence of robot deception behaviors. Across conditions, we found that the most common types of justifications defined a normatively desirable outcome or goal of the robot’s deception, be it to mitigate emotional harm, keep a property safe or to enhance the social bond between the human and robot. These justifications seem to align with Isaac and Bridewell’s (Isaac and Bridewell, 2017) formulation of the role of a deceptive behavior, which is to mislead or obfuscate signals and cues in service of a more desirable goal that the robot aims to achieve, prioritized over transparency or honesty in the human-robot interaction. Participant responses show that they understand justifications as reasons that make the deception defensible, because the overriding goal is more desirable than the normally operative goal of being truthful.

### 3.6 RQ3: do humans perceive other entities (e.g., programmers, designers) as also engaging in deception when a robot commits a deceptive behavior?

The aim of RQ3 was to determine the frequency in which participants identified another entity (besides the robot) as being deceptive in each deception condition.

#### 3.6.1 External state deception

**FIGURE 3:**
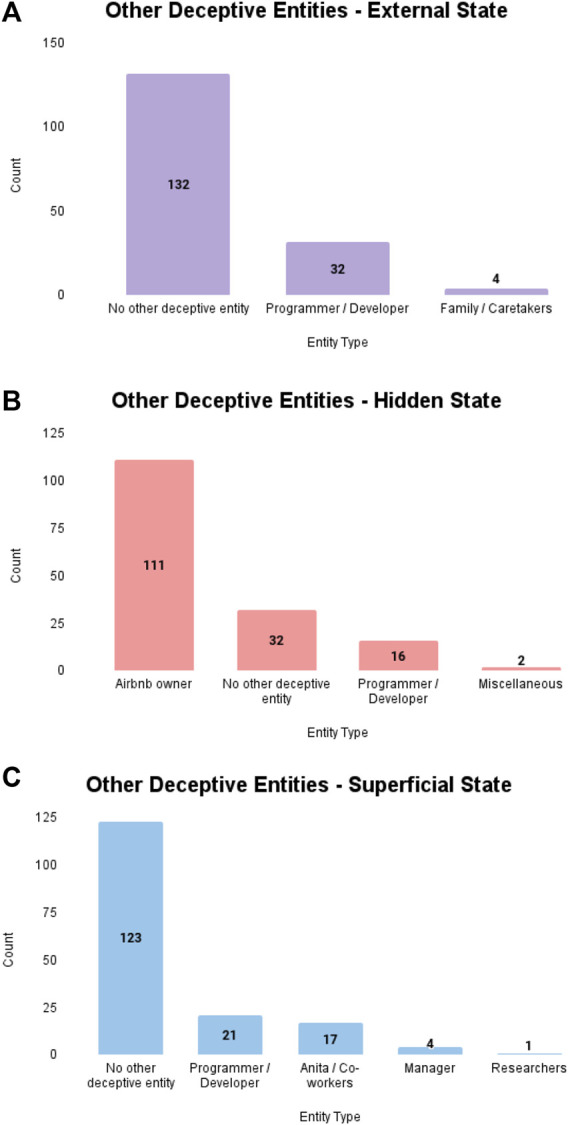
Additional deceptive entities identified by participants in each condition. **(A)** Other deceptive entities identified by participants in external state condition. **(B)** Other deceptive entities identified by participants in hidden state condition. **(C)** Other deceptive entities identified by participants in superficial state condition.

One hundred and thirty-two participants (N = 132, 78.6%) indicated that no other entity besides the robot engaged in external state deception in the scenario. Of the few participants that did reference another entity that engaged in deception in the scenario (see Figure 3A), 36 referenced a programmer or developer and 4 referenced Maria’s family or an undetermined individual that provided Maria with the robot as a caretaker.

#### 3.6.2 Hidden state deception

Only 32 participants (N = 32, 19.9%) indicated that no other entity besides the robot engaged in hidden state deception in the scenario. Of the participants that referenced another entity engaging in deception (see Figure 3B), 111 referenced the owner or manager of the property, 16 referenced the programmer or creator of the robot, and 2 participants referenced other actors such as the renters or the intruders.

#### 3.6.3 Superficial state deception

One hundred and twenty-three participants (N = 123, 75.3%) indicated that no other entity besides the robot engaged in deception in the scenario. Of the few participants that referenced another entity engaging in deception (see Figure 3C), 17 participants referenced Anita or the robot’s co-workers in the home goods store, 21 referenced the programmer or creator of the robot, 4 referenced the manager or employer of the home goods store, and 1 participant referenced the researchers.

Our analysis of participant responses to other entities that committed a deception showed that a majority of participants in the hidden state condition extended the robot’s deception to another entity, mainly the Airbnb owner. In contrast, the majority of participants in the external state and superficial state conditions tended to isolate the deceptive behavior to the robot. Across all conditions, a subset of participants would refer to a programmer or developer as a possible entity that would also be considered deceptive in each scenario.

## 4 Discussion

Although the technology ethics literature has detailed ways in which robot deception could manifest (Danaher, 2020a; Leong and Selinger, 2019; Isaac and Bridewell, 2017; Sætra, 2021) and discussed potential consequences resulting from exposure to these deceptive behaviors (Sharkey and Sharkey, 2021; Turkle et al., 2006; Bisconti and Nardi, 2018), little experimental work has been conducted examining people’s perceptions of these deceptive behaviors. Using a mix of quantitative and qualitative data, our study aimed to provide some of the first experimental evidence on whether robot acts theorized to be deceptive are actually perceived as such. We first briefly summarize the main findings, then turn to their implications for dishonest anthropomorphism, the deception objection debate, and future experiments examining robot deception types and its effects on human-robot interactions.

We examined participant perceptions of the deceptiveness and approval of three types of behavior labeled by Danaher as (1) External state deception (deceptive cues that intentionally misrepresent or omit details from the external world; i.e., lying to an elderly woman that her deceased husband is still alive), (2) Hidden state deception (deceptive cues designed to conceal or obscure the presence of a capacity or internal state the robot possesses; i.e., a robot using its position as a housekeeper to record users without their knowledge), and (3) Superficial state deception (deceptive cues that suggest a robot has some capacity or internal state that it lacks; i.e., a robot expressing pain when moving a heavy object with another person). Our study showed that participants rated hidden state deception as the most deceptive behavior among the three, and they also disapproved of this type of deception much more than of the other two. Further, even though participants viewed both external state and superficial state deception as moderately deceptive, they approved of external state deception considerably more than the superficial state deception.

This study aimed to explore the ”Deception Objection”—the debate regarding whether robots’ superficial states (e.g., emotions, sensations) are problematic, or should (not) be considered deceptive—by examining if participants were unsure about a robot committing superficial state deception behaviors at a higher frequency compared to external state or hidden state behaviors. People were not more unsure about whether a robot’s superficial state was deceptive than they were about the other deception types. This result suggests that superficial state deception may not be as strongly divisive to everyday persons as it is to researchers engaged in the debate.

Beyond people’s approval and perceptions of deceptiveness, we were interested in documenting the kinds of possible justifications participants provided in light of robots’ behavior, and especially whether they referenced superseding norms.

A thematic analysis of participant responses showed that, in each condition, common themes were identified in the justifications that participants provided for the robot’s behaviors. In the external state condition, participants justified the robot’s deceptive behavior as a means of preventing Maria from feeling harm or keeping Maria calm. Participants in the hidden state condition justified the robot’s recording as a form of quality control of its functions or a form of security. In the superficial state condition, participants justified the robot’s expression of emotions as a way to develop social bonds with its co-workers or as a means to advance the research goals of the experimental study in which it was engaged.

In addition to the justification themes identified in the thematic analysis, we found that the frequency with which participants provided a justification for the robot’s deceptive behavior varied between conditions. The majority of participants in the external state condition provided a justification for the robot’s behavior, with a small subset of participants explicitly stating that its behavior could not be justified. In both the hidden state and superficial state conditions, less than half of the participants readily provided a justification for the robot’s behavior. In the superficial state condition, the proportion of participants who explicitly stated that the robot’s behavior was not justifiable was just below the proportion of people who provided a justification. In the hidden state condition, the proportion of participants who explicitly stated that the robot’s behavior was not justifiable was greater than the proportion of people who provided a justification.

We also assessed how many participants identified third parties beyond the robot as involved in the robot’s deceptive behavior and who those third parties were. Results showed that a majority of participants encountering a hidden state deception identified a third party as being deceptive besides the robot in the vignette (most commonly the owner of the rental home and the robot developer). Although participants in the external state and superficial state conditions did extend the robot’s deception to third parties, with the developer of the robot being a common third party identified in each condition, they did so much less frequently than those in the hidden state condition. These results may be evidence that for certain types of deception, the robot is no longer viewed as the main entity committing deception, but rather a “vessel” (Arkin, 2018) by which deception occurs in the service of another entity advancing its goals through the robot.

### 4.1 Perceptions of different deceptive behaviors

The findings from RQ1 and H1 indicate that robots that commit external state deception (e.g., lying) may, in certain contexts, be perceived as committing an acceptable behavior. In fact, the majority of participants readily provided justifications for the robot’s behavior in the external state condition, referencing norms such as sparing a person’s feelings or preventing harm. Thus, people may have inferred that the robot was following human social norms, perhaps even understanding the logic of a “white lie” (Isaac and Bridewell, 2017), trading off a standing norm (not lying) against a superseding norm (e.g., sparing a person’s feelings).

In contrast, people found the hidden state deception (e.g., a robot using its position as a housekeeper to record users without their knowledge) to be highly deceptive and disapproved of it. They may have experienced this type of deception as a “betrayal” (Danaher, 2020a) committed by the robot—or through the robot, as many participants indicated that they felt other parties were involved in the deception. This betrayal, stemming from not disclosing machine-like features (i.e., continuous hidden surveillance) while simultaneously operating in a human-like role (i.e., the robot’s role as a housekeeper), may explain why participants strongly disapproved of the hidden state behavior and viewed it as highly deceptive. These findings provide strong evidence for the negative consequences of such dishonest anthropomorphic designs. If people realize that a robot designed for a certain role has hidden abilities and goals that undermine this role, people may consider discontinuing its use because the robot does not primarily serve them but the interests of a third party.

Exactly who is being served/supported by robots as they are deployed into social spaces is important to consider. Such deployment may represent juxtaposed or potentially competing needs and goals between different “users” of the robots (Phillips et al., 2023). In the hidden state scenario, some people may feel deceived when encountering these robots in their environments, and that deception is evaluated negatively. However, the people who deployed the robots in those environments in the first place may not see them as similarly deceptive and problematic. A real challenge for social robots, then, is determining and potentially balancing “whose goals and needs” are the ones that are justifiable and should supersede in these cases.

### 4.2 Exploring justifications of deceptive behaviors

In this study, we argued that justifications can represent superseding norms as they reference norms that motivated an agent’s actions. Recent work has shown that justifications can repair losses in trust and mitigate blame for other types of social norm violations committed by robots (Malle and Phillips, 2023). Here we ask if forms of deception as a type of norm violation might be similarly justifiable.

Humans may be readily capable of understanding the underlying social and moral mechanisms that drive the pro-social desires for lying and thus are capable of articulating a justification for the robot’s similar behavior in the external state condition. Danaher posited that in cases of external state deception committed by robots, humans will react and respond to the robot’s behavior in a manner similar to a human in a comparable position (Danaher, 2020a).

Participants in the hidden state deception condition provided the smallest proportion of justifications as well as the largest proportion of responses that extended involvement in the deceptive behavior to someone else beyond the robot. These findings suggest that hidden state deception was the most difficult type of deceptive behavior for humans to reconcile and that any superseding norm evoked would need to be powerful in order to compensate for the high disapproval associated with this type of robot behavior, or associated with other humans using robots in this way.

Using robots in this way without disclosing their full capabilities may simply not be justifiable. And for good reasons. These findings seem to mirror reports about strong negative reactions people have to real-world examples of robots in homes that have arguably engaged in similar forms of deception (Guo, 2022). For instance, MIT Technology Review recently reported that when iRobot was testing new software deployments on the Roomba J7 robot vacuums in homes, the robots were capturing sensitive images of people, including of young children, and people using the toilet. Further, those images were being annotated by other people who had been contracted to label them as training data for future software development, and these images were ultimately leaked to social media groups online. iRobot confirmed that these images came from individuals that had agreed to their imagery being captured in user license agreements. Clearly, however, this form of non-disclosure/under disclosure was still problematic for those involved. Onerous user agreements or implicit “opt-in” policies that are likely to come with social robots in the real-world run a real risk of perpetuating forms of deception that people indeed do not agree with, but are subjected to nonetheless.

Participants in the superficial state condition provided justifications for the robot’s behavior of claiming that it was “in pain” while helping to move a heavy object. However, the proportion of participants who explicitly referred to a justification was only slightly more frequent (N = 45) than those who would not justify the robot’s behavior (N = 38). These findings suggest that participants may have issues justifying a robot’s expression of certain internal states in certain interaction contexts depending on the outcome of the interaction and the robot’s perceived goals in expressing that internal state.

In our experiment, the robot introduced in the scenario is operating in the role of a home goods worker. The robot may be viewed as a depiction (Clark and Fischer, 2023) of a home goods worker, capable of completing tasks and interacting with fellow workers in the way a typical human home goods worker may be expected to act. Conflict occurs when the robot is expressing pain and how Anita responds to that pain. In our scenario, the outcome of the robot’s behavior (expressing a superficial state of pain it does not possess) resulted in Anita telling the robot to stop working and telling another co-worker to take the robot’s place. It may be the case that so many participants failed to provide a justification for the robot’s behavior because the outcome of its behavior was that another human had to do its work; and for the false reason that the robot “was in pain,” even though the robot is not capable of feeling pain.

It is possible that participants found the robot’s behavior in the scenario manipulative, whether intentional or not, because the robot’s expression of pain influenced one of the worker’s behavior in a way that led to an inconvenience for another worker. Manipulation of users is one of the arguments critics of emotionally expressive robots point to (Sharkey and Sharkey, 2012; Turkle et al., 2006; Bisconti and Nardi, 2018) as a potential danger in allowing social robots to form emotional bonds with people. Manipulation aside, research has also shown that robots that express emotions can also provide beneficial outcomes such as making people more engaged in collaborative tasks with the robot (Leite et al., 2008) and perceiving the robot as more emotionally intelligent (Jones and Deeming, 2008). It is fair to wonder, then, whether participant approval and justification of the robot’s behavior in superficial state deception is dependent on the way its expression of a superficial state influences human behavior and interaction outcomes, and how beneficial or detrimental those behaviors or outcomes are.

### 4.3 Acknowledgement of other entities engaging in deception

In our analysis of the presence of other entities besides the robot that deceived, participants were much more likely to extend the deception to other entities beyond the robot in the hidden state condition than either the superficial state or external state condition. In the hidden state condition, 85% of participants extended deceptive behaviors beyond the robot, often directly evoking the robot as a machine that was either programmed or directed by a third party to deceive the humans in the vignette. This finding may provide evidence for the claim that some forms of deception committed by robots are less actions that the robot itself chose to take, but rather are a vessel for another agent that is the deceiver.

In the external and superficial state conditions, most participants did not extend the deceptive behavior committed by the robot to other agents. In both conditions, about 75% of participants isolated the deceptive behavior to the robot alone. However, when we examined participant justifications provided in these conditions, many participants also acknowledged that the robot was explicitly programmed (e.g., stating that the behavior could not be justified because the robot is simply a programmed machine), suggesting that some individuals in these conditions similarly acknowledged that there is a third party involved in the robot’s behavior. However, far fewer people in these conditions extended the deception to such third party actors. In the superficial state condition, 62% of participants that believed the robot was programmed did not consider other actors as deceptive in the scenario; in the external state condition, 43% did the same.

The finding that people in our study simultaneously acknowledged that others were involved in the programming of the robot while not extending deception to those people are in line with other discussions in the research community about potentially negative ramifications of using robots as vessels (Arkin, 2018) for humans committing potentially problematic behaviors. For instance, Gratch and Fast (2022) argue that the use of artificial agents represents a form of “indirect action” where one party acts through another agent. Problematically, when people in power deceive or cause harm, they often choose such indirect action because doing so attenuates social pressures that would normally regulate unethical behavior; for instance, it may deflect blame away from the actor and towards the intervening agent. Further, these same researchers have found in experimental studies that artificial agents, like A.I. powered assistants, trigger the same type of attenuation. Specifically, when students were interacting with an A.I. agent that they thought was previously programmed to provide critical, non-empathetic feedback while they were studying for an exam, those students gave less blame towards the previously programmed agent than they did towards an avatar that they believed represented a human responding in real time, providing the same harsh feedback in another room.

These findings highlight the role the developer plays in a human-robot interaction, particularly when the robot commits deceptive behaviors. While not directly involved in the interaction, developers seem to be held responsible by humans exposed to a robot’s deceptive behavior. This finding is important because humans that are exposed to deceptive robot behaviors could extend the deception to developers and the organization that produced the robots. This could potentially lead to a global trust loss in robotics technology.

### 4.4 Limitations

This study provides initial findings in a field of research with previously few empirical studies. However, it comes with numerous limitations. First, the study used text-based narratives as stimuli, and it would be important to expand the methodology to include video-based stimuli and real-time interactions between humans and robots.

Second, we aimed to design scenarios representative of real-world examples of social robots’ deceptive behavior, but each of the three types of deception appeared in only one specific scenario. This way, the scenarios were tailored to their particular deception types, but they varied in other features as well. In future efforts, it would be desirable to develop a larger number of more general scenario templates such that a given scenario template could, with small adjustments, represent each of the three deception types, and each deception type would be represented by multiple scenarios.

Third, the assessment of justifications, though informative because open-ended, afforded no experimental control. Furthermore, it was not the robot that conveyed the justifications but participants who proposed them, so these justifications may or may not be credible when uttered by a robot after committing a deceptive behavior. Future work could develop multiple versions of justifications and other responses (e.g., a weak one, a strong one, referring to norms vs. goals) for each deception type and experimentally assign them to participants, who would then evaluate their persuasiveness and credibility (see Malle and Phillips, 2023). Such work would be needed to determine the effectiveness of justifications for such deceptive behaviors.

### 4.5 Future Directions

Although this work aimed to provide some of the first empirical evidence of the perceptions of different kinds of robot deception in human-robot interaction, we believe this study is the first of many steps towards fully comprehending the nuances of robot deception in human-robot interactions. Future research should look to expand upon the findings from this study by examining whether the justifications uncovered through this experiment could effectively work in mitigating human trust loss and moral judgment towards the robot, especially in cases where robot deception is under the umbrella of dishonest anthropomorphism.

Additionally, future research should expand the findings of this paper by examining deceptive behaviors committed by robots in real world human-robot interactions. Although vignettes are a valuable tool for uncovering initial human perceptions of robot deception, in-lab or real-world studies would allow us to gain even greater insight into how people react to robot deception. Studies with longitudinal designs in which humans are exposed to multiple deceptive acts across a period of time would also provide valuable insight into the effects of deception on human-robot interactions. These experiments could be carried out in environments that mirror the scenarios created for this study, giving researchers a foundation for both a potential interaction that could be tested and serve as a comparison point for what human perceptions of the deceptive act could be.

### 4.6 Conclusion

Our study examined to what degree participants approved of, and actually judged as deceptive, three types of deceptive behavior that Danaher (2020a) argued could all be committed by robots. We then proceeded by examining possible justifications that participants could provide in light of the robot’s behavior and, finally, we analyzed the frequency with which participants extended the deception committed by the robot to other entities.

The contribution of this work is to advance our understanding of deception in human-robot interactions by studying human perceptions of three unique deception types that robots may possess. Primary empirical evidence showed that deceptive behavior types relating to dishonest anthropomorphism (hidden state deception and superficial state deception) are found to be particularly devalued and less likely to be justifiable by people. External state deception, including white lies, might be approved, especially if the deception is in service of a superseding norm. We found that people were not as divisive about the deceptiveness of superficial state deception, a topic considered divisive in the tech ethics literature referenced as the “Deception Objection” debate. Results additionally showed that participants who are exposed to hidden state deception in robots tend to extend the deception to other entities besides the robot, a trend not found in either external state or superficial state deception conditions. This work advances moral psychology in human-robot interactions by exploring the potential consequences of human discovery of robot deception and potential social norms that could be evoked as a trust repair strategy in the face of trust loss and moral judgement.

## Data Availability

The datasets presented in this manuscript and in our Supplementary Materials can be found in the following online repository: https://github.com/arosero98/-Exploratory-Analysis-of-Human-Perceptions-to-Social-Robot-Deception-Behaviors-Datasets.
