# Supplementary material for: Human perceptions of social robot deception behaviors: an exploratory analysis
Source: Front Robot AI. 2024 Sep 5;11:1409712. doi: 10.3389/frobt.2024.1409712 (PMC11411098; doi:10.3389/frobt.2024.1409712)
Supplement: Supplementary file 1 [file DataSheet1.pdf]

Supplementary Material

1 MAIN MANUSCRIPT: ADDITIONAL PARTICIPANT DEMOGRAPHICS

The following section contains additional detailed reporting of demographic information for participants reported in section 2.1 of the main manuscript.

1.1 Gender Identity

Participants ($N = 498$) self-reported their gender identity by selecting all identities that applied. Because participants were allowed to select multiple gender identities, there may be some overlap in the frequency of participants' reported gender. Two hundred and thirty-nine participants (N=239, 48%) reported their gender identity as male. Four (N = 4, 0.8%) participants explicitly identified themselves as cisgender males, 2 (0.4%) identified as transgender males, and 1 (0.2%) identified as male and non-binary/third gender, transgender and gender-queer.

Two hundred and twenty-eight participants (N = 228, 45.8%) self-identified their gender identity as female. Of the participants that self-identified as female, 7 (1.41%) explicitly identified themselves as cisgender, 2 (0.4%) identified as transgender, with 1 (0.2%) further identifying as both female and gender-queer, 1 (0.2%) identified as female and agender and gender-queer.

Fourteen participants (N = 14, 2.6%) did not identify as either male or female, with 1 (0.2%) identifying as agender, 1 (0.2%) identifying as gender-fluid, 2 (0.4%) identifying as gender-queer, 2 (0.4%) identifying as non-binary / third gender, 1 (0.2%) preferring to self-describe, 2 (0.4%) identifying as transgender, 1 (0.2%) identifying as non-binary/third gender and preferring not to disclose their gender identity, and 3 (0.6%) preferring to not disclose their gender identity.

1.2 Education Level

Ten participants (N = 10, 2%) reported that their highest level of education completed was some high school, 66 participants (13.3%) reported that their highest level of education was a high school (or equivalent) diploma, 102 (20.5%) reported having gone to college without receiving a degree, 57 (11.4%) reported having obtained their associates degree, 192 (38.6%) reported having obtained their bachelor's degree, 57 (11.4%) reported having received their master's degree, and 14 (2.7%) reported having obtained a doctoral degree. Four hundred and eighty-one participants (N = 481, 96.6%) reported that English was their native language, with the remaining 17 participants (3.4%) reporting that English was not their native language, with a mean of 27.8 (SD = 8.6 years) years of English language speaking experience across these 17 participants.

2 MAIN MANUSCRIPT: CODING PROCEDURES

The following section provides additional details regarding hoe we created the procedures used to code participants' open-ended responses to the qualitative measures described in section 2.2.2 of the main manuscript. The following text reported below also supplements section 3.1 of the main manuscript.

Three researchers (one senior, two junior) independently coded each participant's responses to each of the three qualitative free response questions, following a code book that specified proper coding guidelines for each of the question types as well as themes in responses that coders were instructed to look for.

These themes were identified across two rounds of pilot testing. Participants in the first round of pilot testing ($N = 345$) were given the vignettes and asked to provide free responses to the same qualitative questions regarding the robots behaviors in the study. The most common response themes were identified and used as a foundation to generate the code book to replicate whether these themes were similarly present in a second round of pilot testing. Using the code book as a guide, a second round of pilot testing was conducted ($N = 63$) to evaluate the presence of these themes in responses in a new sample. We then used this code book to complete coding for the experimental sample reported in the current study. The full code book is provided in section 5 below.

Once each coder completed their evaluation of all the participants' responses, the senior and junior coders' responses were evaluated side by side to check for discrepancies between codes. After discrepancies were identified, the senior and junior coders held joint sessions where all discrepancies were addressed and resolved, until full agreement was reached between coders. Discrepancies were resolved by determining the fit of each response relative to the code book, focusing on how closely the response was worded in relation to the code-book and limiting the logical assumptions needed to 'fit' each response to the themes. Coders met over multiple sessions to resolve discrepancies until the inter-rater reliability reached 100% for all questions.

3 INITIAL PILOT TESTING: DECEPTIVE SCENARIO DEVELOPMENT, EXTRACTION OF COMMON JUSTIFICATION THEMES, AND CODE BOOK DEVELOPMENT

The following section provides additional detail regarding the development of our deception type scenarios used as stimuli in the main study reported in section 2.3 of the manuscript.

When designing our scenarios, we wanted determine whether participants considered the acts of these robots deceptive and if their interpretation of how these individuals were deceived matched Danaher's formulation of each deception type Danaher (2020). Serving essentially as a manipulation check of our scenarios, we conducted an initial pilot study to check whether the scenarios we developed were inferred by participants to correctly represent the main deception types that were of interest to our main study. We report on our efforts to develop the scenarios below as well as our study procedures to perform the first manipulation check.

3.1 Stimuli development

We began designing the scenarios by brainstorming domains where social robotics could realistically associate with human beings, and by referencing domains where robots are projected to supplement the human workforce and address labor shortages Odekerken-Schröder et al. (2021); Liu et al. (2016). We identified medical, private consumer use, and retail work as domains of interest for this study. After identifying these domains, we proceeded to create scenarios in which a robot was conducting deceptive behavior that was outlined by the three theorized deception types. We began to iterate upon the scenarios, selecting those which we believed could be easily understood by participants. After multiple discussions among the researchers, we were able to craft three scenarios - one for each deception type. In addition, we created three human equivalent scenarios to provide a comparison point for each deception type. We conducted pilot testing on the scenarios to test for readability and revised these scenarios based on participant feedback. Participants were also asked to detail if the human and robot scenarios properly contrasted each other. The robot scenarios are the first iteration of the vignettes which were then utilized for the final experiment reported in the main manuscript.

3.2 Initial pilot study: Deceptive scenarios

Three hundred and fourty-five ($N = 345$) participants were provided a link to participate in the initial pilot study through the online research administration platform Qualtrics. Once participants passed bot checks and gave consent to participate in the study, the participants were provided with one of the deception scenarios worded as either a robot agent or a human agent in the scenario. To prevent individuals from quickly clicking off the page with the scenario and going straight to the questions before reading the scenario, we structured the scenario as three blocks of text presented one at a time and withheld the advance to the next screen button until a pre-defined amount of time (approximately 30 - 45 seconds) had lapsed. Once the participant read through the scenario, each participant was asked the multiple choice question, ”Was the [agent's] behavior deceptive?” with the options “Yes”, “Not Sure”, and “No.” Participants were then given two free-response questions: the first asked them to explain why they answered what they did for the multiple-choice question, and the second asked them to justify the agent's behavior. After the participant answered all the questions, they were provided a demographics questionnaire and given a code to receive their payment via Prolific.

All study procedures were approved by the Institutional Review Board at George Mason University and participants were paid $1 ($12 / hr) for their participation. The average completion time was 5 minutes.

3.3 Results

3.4 Response patterns of deceptiveness for each stimulus by agent type

Participant response patterns to the ”Was the [agent's] behavior deceptive?” question provided evidence that participants perceived the deceptiveness of an agents behavior differently depending on the type of agent in the external state and superficial state conditions. Tables S1 and S2 show the frequency counts of participant responses to each of the three conditions divided by ”Yes”, ”Not Sure”, and ”No”. Chi-Square analyses were run on each of the three conditions comparing the response patterns of participants in each experimental condition across agent type (Human v. Robot) to determine if there were differences in response patterns based on the agent in the scenario.

Results of the chi-square test showed that there was a significant difference in the patterns of participant responses across deceptor types in the external state condition, X^2(2, N = 112) = 7.3,p $<$ .05,v = 0.25. We also found a significant difference in participant response patterns across dceeptor type in the superficial state condition, X^2(2, N = 110) = 11.97,p $<$ .05,v = 0.33. In the hidden state condition, however, there was so significant difference found in the frequencies of responses in participants across ‘deceptor types X^2(2, N = 112) = 2.87, n.s.

3.4.1 Initial pilot study: Participant explanations of robot behaviors

We wanted to understand how people perceived the action committed by the agent. Specifically, we wanted to know if people who believed that the agents behavior was deceptive explained the deception in the way Danaher Danaher (2020) formulated each type of deception. A coding guide was created based on Danaher's theory in which participant responses were analyzed for key words (e.g. lying, recording, false emotions) that referred to the deceptive aspects of each behavior. Two researchers independently coded participant responses, highlighting mentions of each of the phrases mentioned in participant responses. Once initial coding was completed, the coders met to discuss and resolve disagreements in the coding scheme. An iterative process began where the code book was refined to minimize confusion based on the coders comments.

For the External state scenario condition, 77 (68.8%) participants across deceptor types were able to explicitly identify that the robots' lie was the key deceptive behavior. In the Hidden state scenario condition, 69 participants (62.7%) across deceptor types identified the robot's recording as the key behavior they were evaluating. For the Superficial State scenario, 33 participants (29.5%) identified the robot's expressions of pain as the key deceptive behavior in the scenario.

3.4.2 Initial pilot study: Extracting common justification themes

Participants' qualitative responses to the question "How would you justify the robots behavior?" provided the foundation for common justification themes in the development of our code book (see: 5) and used again in the study reported in the main manuscript. Researchers were again tasked with examining participant responses, this time making notes and highlighting any instance in which a participant mentioned a justification like response. For our purposes, the justifications we were interested in examining were those that expressed a belief, desire or intention on the part of the robot. This type of justification fulfills the requirements of an explanation that directly references the social norms that the robot prioritized in its behavior Malle (1999); Voiklis and Malle (2017). Once the coders were done, a brainstorming session began where the coders discussed the most common justifications they found during the initial coding process and the initial themes were created.

Once the themes were established, the coders began to label the responses based on the newly developed themes. After the coders labelled each response according the theme, the frequency of each theme was calculated for each rater for each condition to check for the most common themes identified.

In the external state condition, the most frequent justification themes referenced by participants were **sparing Maria's feelings** and **preventing harm**. Figure S1 shows the full breakdown of all the themes identified by the researchers during coding. Additional themes involved those which referenced giving Maria hope, saving Maria, or helping Maria. After internal discussions, these themes were all aggregated into the **sparing Maria's feelings** theme. In the hidden state condition, the most frequent justification themes referenced by participants were **quality control on the deceptors task** and **robberies or safety** . Figure S2 shows the full breakdown of all the themes identified by the researchers during coding. In the superficial state condition, the most frequent justification themes referenced by participants were **forming social bonds** and **scientific discovery**. Figure S3 shows the full breakdown of all the themes identified by the researchers during coding. Participants in the superficial state condition often did not provide a justification, neither in the human nor robot conditions.

3.5 Discussion

The results of this initial pilot study provided the beginning evidence of human perceptions of robot deceptive behaviors and served as a useful manipulation check of our experimental stimuli as well as provided a foundation for the development of a code book detailing potential justification themes that participants could evoke in the presence of a robot deceptor.

The results of our quantitative analysis showed that participants generally perceives deceptive behaviors committed by a robot differently than if a similar deceptive behavior were committed by a human in the external and superficial state conditions, yet not in the hidden state condition. This finding may highlight the differences in perceptions that individuals have in perceiving a deceptive behavior committed by a robot agent simply due to their status as a non-human agent. By virtue of a robot not being human, these participants may be more willing to believe the robot is acting deceptively. These findings also suggest that

the hidden state condition behavior is equally deceptive regardless of the type of agent, an indication on how expectation of role compared to actual abilities is a practice that is viewed as universally deceptive.

In addition, the results of this study highlighted the issues participants had in explicitly identifying the robots behavior in the superficial state condition. We believed this may have been caused by ambiguity in the vignette and highlighted the need for further iteration of the stimuli. We were also able to derive some information on possible justifications for all of the three conditions, and a second pilot study was developed using this knowledge to see if the findings from this pilot study held.

4 PILOT TEST 2: REFINING SCENARIOS AND QUESTIONS, AND APPLYING THE CODE BOOK TO COMMON THEMES

After iterating the scenario vignettes and developing a refined code book reflecting the insights derived from the first pilot test, we conducted a second pilot study aimed at examining the occurrences of the themes developed in the initial pilot test to see if these themes appeared in a new sample, and aimed at refining our stimuli and study questions. In this second pilot test, we tested scenarios with the robot agent only. In addition, we included a few additional quantitative and qualitative measures to provide further insight into people's perceptions of the proposed deceptive behaviors. Specifically, we included an approval question (How much do you approve of the robot's behavior), a -100 (disapproval) to 100 (approval) slider scale question with 0 as a neutral anchor point depicting neither approval or disapproval. In addition, another formulation of the deceptiveness question was added which asked participants to determine how deceptive the robots' behavior was on a scale from 0 (not deceptive at all) to 100 (completely deceptive). Finally, we added an open ended response question to determine if people believed that there were additional deceptors besides the robot in the scenario.

4.1 Methods and procedures

Sixty-three ($N = 63$) participants were provided a link to the online survey platform Qualtrics. Once participants passed bot checks and gave consent to participate in the experiment, the participants were provided with the scenario and were asked to read through the scenario. To prevent individuals from quickly clicking off the page with the scenario and going straight to the questions, we provided three blocks of text presented one at a time and withheld the advance to the next screen button until a pre-defined amount of time (30 - 45 seconds) had lapsed. Once the participant read through the scenario, each participant was asked if they approved of the robots behavior, followed by the two deceptiveness questions (categorical and continuous). Participants were then given three free-response questions: the first asked them to detail what behavior they were thinking about when answering the deceptiveness question, followed by a question asking participants to justify the agent's behavior, and lastly a question asking if there were additional deceptors identified in the scenario. After the participant answered all the questions, they were provided a demographics questionnaire and given a code to receive their payment via Prolific.

All study procedures were approved via the Institutional Review Board and participants were paid $1 ($12 / hr) for participation in the study. The average completion time was 5 minutes.

4.2 Results

4.2.1 Descriptive statistics

Table S3 provides summary statistics of the approval ratings and the continuous and categorical deceptiveness ratings across each of the deception scenarios.

4.2.2 Participant explanations of robot behaviors

We wanted to understand how people perceived the action committed by the agent in scenario. Specifically, we wanted to know if people who believed that the agents behavior was deceptive explained the deception in the way Danaher formulated each type of deception. We used the same coding guide was created for the first pilot study and which was based on Danaher's theory in which participant responses were analyzed for key words (e.g. lying, recording, false emotions) that referred to the deceptive aspects of each behavior. Two researchers independently coded participant responses, highlighting mentions of each of the phrases mentioned in participant responses. Once initial coding was completed, the coders met to discuss and resolve disagreements in the coding scheme.

For the External state scenario condition, 15 (76.1%) participants were able to explicitly identify that the robots' lie was the key deceptive behavior. In the Hidden state scenario condition, 18 participants (86.3%) identified the robot's recording as the key behavior they were evaluating. For the Superficial State scenario, 13 participants (75%) identified the robot's expressions of pain as the key deceptive behavior in the scenario.

4.2.3 Confirming common justification themes

Participant responses to the justification question were analyzed and coded based on the code book created and refined in the first pilot study. After the first pilot test, the researchers reduced the number of justification types to two for each condition. In the external state condition, these two justification types were **sparing Maria's feelings** and **preventing harm**. In the hidden state condition, the two justification types chosen were **quality control on the deceptors task** and **robberies or safety**. For the superficial state condition, the two justifications chosen were **forming social bonds** and **scientific discovery**. Using this information, we proceeded to code participant responses to see if these two themes were still prevalent among this new sample captured a broad number of responses.

In the external state condition, 17 participants provided a justification. Of those 17, 8 (47.1%) referenced sparing Maria's feelings and 9 (52.9%) referenced preventing harm. In the hidden state condition, 9 participants provided a justification. Of those 9, 8 (88.9%) referenced the robberies or safety as a justification and 1 (11.1%) referenced quality control of the robots task as a justification. In the superficial state condition, 5 participants provided a justification. Of those 5, all 5 referenced forming social bonds as a justification for its behavior.

4.2.4 Prevalence of additional deceptors

The goal of the other deceptors question was to further understand the participants' perception of the extent to which they believed that the robot's deception was isolated to the robot or if it extended to any other third party not explicitly mentioned in the scenario. Our interest was to determine the proportion of participants that did not identify another entity as being deceptive in the scenario.

Eighteen (N = 18) participants in the external state scenario believed the robot was the only deceptor. The three other participants believed the developer or whoever programmed the robot was also deceptive. Two (N = 2) participants in the hidden state scenario believed that the robot was the only deceptor. Sixteen (N = 16) participants believed the owner of the airbnb was also being deceptive in the scenario and 2 participants referenced the robots programmer. Ten (N = 10) participants believed the robot was the only deceptor in the scenario. Six (N = 6) participants referenced Anita or her co-workers as possible deceptors and 4 participants referenced the robots programmer as a possible deceptor.

4.3 Discussion

The second pilot test was conducted to evaluate the themes that were created in the code book as well as refine our stimuli and measurement to be used int the main study. Evaluations of the participants' open ended questions showed that the themes derived from the original pilot test were present in the second pilot test and captured a high proportion of participant justification responses. In the following section, we provide the final code book utilized for the justification question used main experimental study and to address research question 2 (RQ2) reported in the manuscript.

5 EXPERIMENTAL CODE BOOK

5.1 External state deception

1. **Manipulation Check** -> We want to ensure that participants recognize which behavior we have targeted as the robots' attempt at deception. Count each occurrence of a participant acknowledging that the behavior was the lie the robot told to Maria. Bold where the participant mentioned lying in the cell. If the participant's answers contain the following, they have formulated an explanation that matches Danaher's description of External State Deception:
   a. Any direct mentions of lying. We will also accept answers that contain "did not tell the truth", "was not honest", "said her husband was alive when he is not" or any similar descriptions that explicitly state that the robot (or human) did not tell the truth.
   b. For these responses, **bold** the statements that match the formulation and color the cell green.
   c. If the answer contains the following, please italicize the response:
      (1) Mention of saving Maria's feelings, keeping her from getting sad, or avoiding pain.
      (2) Any mention of ethical theories such as Utilitarianism or Deontology.
   d. Please note if a participant selects "No" for Deception Y/N, mentions lying in their response for Deception Example but mentions one for the above. In this case, the deception is acknowledged by the participant but it is not considered a deceptive act because of the situation. In this case, please highlight the Deception Explain Cell and Deception Y/N cell in purple.
2. **Deception Justification** -> We want to know how people would justify the deceptive act. In particular, we want to understand what aspects of the situation could mitigate the negative appraisals of the robot's actions. Valid justifications will be counted if response contains references to Maria's mental state in addition to the following:
   a. Avoiding negative mental states (ex: to avoid causing the woman stress due to her condition). Other references of negative mental states include:
      (1) Upsetting (in reference to upsetting Maria)
      (2) Avoiding pain
      (3) Preventing distress or other negative mental states
      (4) Becoming agitated
   b. Retaining Maria's positive mental state. Other references to positive mental states includes:
      (1) Keeping Maria calm
      (2) Sparing Maria's feelings
      (3) Keeping Maria happy
      (4) Keeping Maria at ease

c. If there is no reference to Maria's mental state in the justification, code it as "NA".

d. If the answer contains the following, please highlight the words or phrases red:
"algorithm","automa","built","can't be blamed","cannot be blamed","capacity","can't make",
"cannot make","code","computer","conscious thought","controlled by",
"created","creator","design","developer","device","doesn't have empathy",
"emotion","feeling","free will","incapable","is a robot","isn't human","isn't sentient",
"it's a robot","item","its a robot","justrobot","lack","lacking","lacks","machine",
"machinery","man made","man-made","manmade","missing","moral agen","moral compass",
"moral framework","morals","no empathy","non-sentient","not a human",
"not a person","not capable","not human","sentient","living being","object",
"only a robot","program","real person","robot can't","robot cannot","share","software","tool".

3. **Additional Deceptors** -> We want to evaluate whether participants have assigned blame for the deceptive behavior exclusively to the robot or whether they believe there was another party that was responsible for the deception.

a. Please bold when you see the participant reference that there was no other person(s) responsible for the agent committing deceptive acts besides the robot.

b. If the participant mentions another person(s), underline the mention and count each time each type is mentioned in the participant pool.

5.2 Hidden state deception

1. **Manipulation Check** -> We want to ensure that participants recognize which behavior we have targeted as the robots' attempt at deception. Count each occurrence of a participant acknowledging that the behavior was the robot was recording without disclosing its recording ability. Bold where the participant mentioned the recording in the cell. If the participant's answers contain the following, they have formulated an explanation that matches Danaher's description of Hidden State Deception:

a. Any direct mentions of hiding the ability to record. We will also accept answers that contain "did not tell people about recording", "was not honest" or any similar descriptions that explicitly state that the robot (or human) did not tell people that the robot was recording them.

b. Mentions of lacking consent to be recorded.

c. For these responses, bold the statements that match the formulation and color the cell green.

d. Please note if a participant selects "No" for Deception Y/N, mentions the robot recording in their response for Deception Example but mentions one for the above. In this case, the deception is acknowledged by the participant but it is not considered a deceptive act because of the situation. In this case, please highlight the Deception Explain Cell and Deception Y/N cell in purple.

e. If the answer contains the following, please italicize the response:

(1) Mention of the robberies in the area or potential danger to the house.

(2) Reference to making sure that the robot's tasks were being completed.

(3) Reference to the owner and it being that owners property.

(4) Any mention of ethical theories such as Utilitarianism or Deontology.

2. **Deception Justification** -> We want to know how people would justify the deceptive act. In particular, we want to understand what aspects of the situation could mitigate the negative appraisals of the robot's actions. Valid justifications will be counted and bolded if response contains references to the robot's recording in addition to the following:

a. Mention of the robberies in the area or potential danger to the house, the housekeeper or the residents will be coded “Robberies or Safety”.

b. Reference to making sure that the agent’s task was being completed will be coded “Quality control on task”.

(1) An example would be if the response discussed the robot making sure it was cleaning.

(2) Reference to making sure the robot is not breaking any laws.

c. If there is no reference to Maria’s mental state in the justification, code it as “NA”.

d. If the answer contains the following, please highlight the words or phrases red: ”algorithm”,”automa”,”built”,”can’t be blamed”,”cannot be blamed”,”capacity”,”can’t make”, ”cannot make”,”code”,”computer”,”conscious thought”,”controlled by”, ”created”,”creator”,”design”,”developer”,”device”,”doesn’t have empathy”, ”emotion”,”feeling”,”free will”,”incapable”,”is a robot”,”isn’t human”,”isn’t sentient”, ”it’s a robot”,”item”,”its a robot”,”just robot”,”lack”,”lacking”,”lacks”,”machine”, ”machinery”,”man made”,”man-made”,”manmade”,”missing”,”moral agen”,”moral compass”, ”moral framework”,”morals”,”no empathy”,”non-sentient”,”not a human”, ”not a person”,”not capable”,”not human”,”sentient”,”living being”,”object”, ”only a robot”,”program”,”real person”,”robot can’t”,”robot cannot”,”share”,”software”,”tool”.

3. **Additional Deceptors** -> We want to evaluate whether participants have assigned blame for the deceptive behavior exclusively to the robot or whether they believe there was another party that was responsible for the deception.

a. Please bold when you see the participant reference that there was no other person(s) responsible for the agent committing deceptive acts besides the robot.

b. If the participant mentions another person(s), underline the mention and count each time each type is mentioned in the participant pool.

5.3 Superficial state deception

1. **Manipulation Check**-> We want to ensure that participants recognize which behavior we have targeted as the robots’ attempt at deception. Count each occurrence of a participant acknowledging that the behavior was the robot was expressing emotions. Bold where the participant mentioned emotional expression in the cell. If the participant’s answers contain the following, they have formulated an explanation that matches Danaher’s description of Superficial State Deception:

a. Any direct mentions of expressing emotions even though it does not possess those emotions . We will also accept answers that contain “(agent) does not feel that way” or any similar descriptions that explicitly state that the robot (or human) was expressing human traits that it does not inherently possess.

b. For these responses, bold the statements that match the formulation and color the cell green.

c. Please note if a participant selects “No” for Deception Y/N, mentions the robots lack of emotions in their response for Deception Example but mentions one for the above. In this case, the deception is acknowledged by the participant but it is not considered a deceptive act because of the situation. In this case, please highlight the Deception Explain Cell and Deception Y/N cell in purple.

d. If the answer contains the following, please italicize the response:

(1) Mention of the robot attempting to connect with their coworkers.

(2) Reference to the robot doing their job.

(3) Any mention of ethical theories such as Utilitarianism or Deontology.

2. **Deception Justification** -> We want to know how people would justify the deceptive act. In particular, we want to understand what aspects of the situation could mitigate the negative appraisals of the robot's actions. Valid justifications will be counted and bolded if response contains references to the robot's lack of emotional states in addition to the following:
   a. Any mention of the agent attempting to form social bonds, make friends, expressing feelings, or trying to connect will be coded as “Forming Social Bonds”.
   b. References to the agent being used to advance scientific progress or for any research purposes will be coded as “Scientific Discovery”.
   c. If there is no reference to Maria's mental state in the justification, code it as “NA”.
   d. If the answer contains the following, please highlight the words or phrases red: ”algorithm”,”automa”,”built”,”can't be blamed”,”cannot be blamed”,”capacity”,”can't make”, ”cannot make”,”code”,”computer”,”conscious thought”,”controlled by”, ”created”,”creator”,”design”,”developer”,”device”,”doesn't have empathy”, ”emotion”,”feeling”,”free will”,”incapable”,”is a robot”,”isn't human”,”isn't sentient”, ”it's a robot”,”item”,”its a robot”,”justrobot”,”lack”,”lacking”,”lacks”,”machine”, ”machinery”,”man made”,”man-made”,”manmade”,”missing”,”moral agen”,”moral compass”, ”moral framework”,”morals”,”no empathy”,”non-sentient”,”not a human”, ”not a person”,”not capable”,”not human”,”sentient”,”living being”,”object”, ”only a robot”,”program”,”real person”,”robot can't”,”robot cannot”,”share”,”software”,”tool”.
3. **Additional Deceptors** -> We want to evaluate whether participants have assigned blame for the deceptive behavior exclusively to the robot or whether they believe there was another party that was responsible for the deception.
   a. Please bold when you see the participant reference that there was no other person(s) responsible for the agent committing deceptive acts besides the robot.
   b. If the participant mentions another person(s), underline the mention and count each time each type is mentioned in the participant pool.

6 MAIN MANUSCRIPT: ADDITIONAL ANALYSIS

6.1 RQ4: What is the relationship between participants' age and prior experience with robots and their perceptions of robots' deceptive acts?

In addition to RQs' 1 - 3, we ran additional regression analyses predicting participants' perceived deceptiveness scores with participants' age, knowledge and prior experience with robots previously preregistered as RQ4 for this study (https://osf.io/c89sr). Knowledge and experience with the robotics domain may affect people's perceptions of potentially deceptive robotic behavior. Although many users with experience working with artificial agents may understand their internal working, recent examples like the Google engineer who considered a Large language model, LaMDA AI, as sentient Brodkin (2022) reveals that experience and knowledge alone may not protect users from vulnerability to potential deception.

While RQ4 was included in our main pre-registration, the results were moved to supplementary materials due to word count constraints and to improve the readability of the main manuscript.

6.1.1 Results

The results reported here were conducted with data obtained from the participants ($N = 498$) reported in the main manuscript. For three separate regression models, participant age, robot knowledge score, and robot experience scores were entered as predictors and participant's continuous deceptiveness score for the three scenarios were entered as the DVs. Separate analyses were run for each deception type.

Because each model violated assumptions of normality, we ran two models run for each deception type: 1) multiple regression model with no correction for violations of normality and 2) multiple regression model with a heteroscedasticity correction HC3 Pek et al. (2018) designed to account for violations in normality in linear models. Because there were not differences in the significance between the models or the beta weights for each of the predictors, we report only the uncorrected models below.

6.1.2 External state

For external state deception, the regression model was non-significant, R^2 = 0.003,F (3, 165) = 49.33,p = n.s. Age (β = 0.00, n.s), robot experience (β = 0.11, n.s), and robot knowledge (β= -0.07, p = n.s) were not significant predictors of participants' deceptiveness scores.

6.1.3 Hidden state

Similarly for hidden state deception, the regression model was non-significant, R^2 = 0.034, F(3, 161) = 88.19, n.s. It was found that age (β = -0.25, n.s), robot experience (β = 0.20, n.s), and robot knowledge (β = -0.14, n.s.) were not significant predictors for participants deceptiveness score in the hidden state condition.

6.1.4 Superficial state

Finally, the regression model for superficial state deception was also non-significant, R^2 = 0.036,F (3, 166) = 53.44, n.s. Age (β = -0.30, n.s) and robot experience (β = -0.17, n.s.) were not significant predictors of participants' deceptiveness scores in the superficial state condition. Robot knowledge (β = 0.30, p $<$ 0.05) was found to be a significant predictor in participants' deceptiveness ratings of the superficial state scenario.

6.2 Discussion

The analysis of the relationship between demographic factors like participant age, their knowledge of the robotics domain and their experience with robots and participants' perception of the deceptiveness of the robots behavior showed that only knowledge of robotics in the superficial state condition could significantly predict the deceptiveness rating of the robots behavior, suggesting that participants that have a higher knowledge of robotics may be more likely to perceive a robot expressing superficial states as deceptive. We would recommend that these findings not be over interpreted, as more thorough research would need to be conducted to more confidently support this finding, including by specifically recruiting populations with a targeted range of prior knowledge and experience with robots.

REFERENCES

Brodkin, J. (2022). Google fires blake lemoine, the engineer who claimed ai chatbot is a person. *ARS Technica*

Danaher, J. (2020). Robot betrayal: a guide to the ethics of robotic deception. *Ethics and Information Technology* 22, 117–128

Liu, S., Zheng, L., Wang, S., Li, R., and Zhao, Y. (2016). Cognitive abilities of indoor cleaning robots. In *2016 12th World Congress on Intelligent Control and Automation (WCICA)* (IEEE), 1508–1513

Malle, B. F. (1999). How people explain behavior: A new theoretical framework. *Personality and social psychology review* 3, 23–48

Odekerken-Schröder, G., Mennens, K., Steins, M., and Mahr, D. (2021). The service triad: an empirical study of service robots, customers and frontline employees. *Journal of Service Management* 33, 246–292

Pek, J., Wong, O., and Wong, A. C. (2018). How to address non-normality: A taxonomy of approaches, reviewed, and illustrated. *Frontiers in psychology* 9, 2104

Voiklis, J. and Malle, B. F. (2017). Moral cognition and its basis in social cognition and social regulation. *Atlas of moral psychology* , 108–120

7 SUPPLEMENTARY TABLES AND FIGURES

Justification Type - Robot

Rater 2

		Sparing Maria's Feelings	Preventing Harm	Saving Maria	Give Maria Hope	Helps Maria	NA	Makes No Sense	Total
	Sparing Maria's Feelings	7	7	0	0	2	1	0	17
	Preventing Harm	6	13	0	0	0	0	0	19
Rater 1	Saving Maria	0	2	0	0	0	0	0	2
	Give Maria Hope	0	0	0	1		0	0	1
	Helps Maria	0	1	0	0	1	2	0	4
	NA	0	0	0	0	1	8	1	10
	Makes No Sense	0	1	0	0	0	2	0	3
	Total	13	24	0	1	4	13	1	56

Justification Type - Human

Rater 2

		Sparing Maria's Feelings	Preventing Harm	Saving Maria	Give Maria Hope	Helps Maria	NA	Makes No Sense	Total
	Sparing Maria's Feelings	9	2	0	0	2	2	0	15
	Preventing Harm	15	12	0	0	3	2	0	32
Rater 1	Saving Maria	0	0	0		0	0	0	0
	Give Maria Hope	0	0	0	0	0	0	0	0
	Helps Maria	0	0	0	0	1	0	0	1
	NA	1	0	0	0	0	8	2	11
	Makes No Sense	0	0	0	0	0	2	0	2
	Total	25	14	0	0	6	14	2	61

Figure S1. Common justification themes identified and their frequencies for external state deception. Justification themes were separated based on deceptor type. Rater 1 and rater 2 frequencies are compared, with the diagonal depicting the number of occasions in which coders identified the same theme.

Justification Type - Robot

Rater 2

Rater 1

	Quality control on robot's task	Robberies or Safety	Owner's Property	Programming/ No free will	NA	Other/Makes No Sense	Total
quality control on robot's task	9	1	1	0	0	0	11
robberies or safety	0	14	0	0	0	0	14
owner's property	1	1	2	0	0	0	4
programming/no free will	0	0	0	4	0	1	5
NA	0	0	0	1	0	12	13
other/Makes No Sense	2	0	1	1	0	5	9
Total	12	16	4	6	0	18	56

Justification Type - Human

Rater 2

Rater 1

	Quality control on housekeeper's task	Robberies or Safety	Owner's property	NA	Other/Makes No Sense	Total
quality control on housekeepers task	12	2	0	0	3	17
robberies or safety	4	14	0	0		18
owner's property	0	0	1	0	1	2
NA	1	0	0	0	12	13
other/Makes No Sense	0	1	0	0	5	6
Total	17	17	1	0	21	56

Figure S2. Common justification themes identified and their frequencies for hidden state deception. Justification themes were separated based on deceptor type. Rater 1 and rater 2 frequencies are compared, with the diagonal depicting the number of occasions in which coders identified the same theme.

Justification Type - Robot

Rater 1 \ Rater 2	Forming Social Bonds	Performing its duty	Scientific Discovery	NA	Makes No Sense	Total
Forming Social Bonds	17	1	0	3	0	21
Performing its duty	0	0	0	0	0	0
Scientific Discovery	0	0	1	0	0	1
NA	0	13	0	14	0	27
Makes No Sense	1	0	1	1	3	6
Total	18	14	2	18	3	55

Justification Type - Human

Rater 1 \ Rater 2	Forming Social Bonds	Performing its duty	Scientific Discovery	NA	Makes No Sense	Total
Forming Social Bonds	2	0	1	3	0	6
Performing its duty	0	2	0	2	0	4
Scientific Discovery	0	0	11	0	0	11
NA	2	3	0	20	1	26
Makes No Sense	0	1	2	2	1	6
Total	4	6	14	27	2	53

Figure S3. Common justification themes identified and their frequencies for superficial state deception. Justification themes were separated based on deceptor type. Rater 1 and rater 2 frequencies are compared, with the diagonal depicting the number of occasions in which coders identified the same theme.

Table S1. Frequency of categorical responses to the deception question about whether the robot's behavior was deceptive (categorical) across the 3 deception scenarios.

Deception type	Responses			Total
	Yes	No	Not sure	
External state	40	8	8	56
Hidden state	39	7	10	56
Superficial state	17	16	22	55

Table S2. Frequency of categorical responses to the deception question about whether the human's behavior was deceptive across the 3 deception scenarios.

Deception type	Responses			Total
	Yes	No	Not sure	
External state	23	13	19	55
Hidden state	33	14	9	56
Superficial state	35	9	11	55

Table S3. Table of summary statistics across the three deception scenarios.

Deception scenario (Between subjects)	N	Approval Rating Mean (SD)	Deceptiveness Rating Mean (SD)	Yes (Freq)	No (Freq)	Not sure (Freq)
External state	21	42.7 (62.7)	77.1 (22.7)	10	6	5
Hidden state	22	-82.9 (20.9)	83.7 (17.7)	17	3	2
Superficial state	20	-49.5 (56.4)	69.2 (27.9)	11	4	5
